# Patient-Centred and Daily Life-Oriented Botulinum Toxin Treatment for Stroke Survivors with Upper Extremity Spasticity—Effects and Practical Aspects

**DOI:** 10.3390/jcm14238339

**Published:** 2025-11-24

**Authors:** Sybille Roschka, David Punt, Thomas Platz

**Affiliations:** 1 School of Sport, Exercise and Rehabilitation Sciences, University of Birmingham, Birmingham B15 2TT, UK; t.d.punt@bham.ac.uk; 2 BDH-Klinik Greifswald, Institute for Neurorehabilitation and Evidence-Based Practice, University of Greifswald, 17491 Greifswald, Germany; thomas.platz@uni-greifswald.de; 3 Neurorehabilitation Research Group, University Medical Centre Greifswald, 17475 Greifswald, Germany

**Keywords:** stroke, spasticity, upper extremity, botulinum toxin, activities of daily living, patient reported outcome measures, patient-centred

## Abstract

**Background/Objectives**: To investigate the impact of a routine botulinum toxin type A (BoNT-A) injection in combination with outpatient therapy on the daily activities of stroke survivors with upper extremity spasticity and to facilitate patient-centred assessment focusing on individual needs during daily life. **Methods**: Design: Observational study across one treatment cycle (3 months). Setting: Spasticity outpatient clinic of a neurorehabilitation hospital in Germany. Participants: Adult stroke survivors (*n* = 27) with upper extremity spasticity receiving routine BoNT-A treatment. Interventions: Participants received one BoNT-A injection and outpatient therapies as part of their routine management. Augmented assessment was conducted directly before the injection (T0), and at 4 to 6 weeks (Tmax1) and 12 to 14 weeks (T2) following the injection. Main outcome measures: The Canadian Occupational Performance Measure (COPM), Goal Attainment Scaling (GAS), and Arm Activity Measure (ArmA). Secondary outcome measures: The Resistance to Passive Movement Scale (REPAS), Motricity Index (MI), SF-12v2 Health Survey (SF-12v2), Global Clinical Impression (GCI), and importance of and satisfaction with the BoNT-A treatment. **Results**: Performance of individually selected daily activities and satisfaction with their performance (COPM), passive care tasks (ArmA, part A), and resistance to passive movement (REPAS) significantly improved from T0 to Tmax1. Improvements largely remained at T2. Individual goals were all set at the activities and participation levels of the International Classification of Functioning, Disability and Health. These improved for 75% of participants and were fully attained by 33.3% at Tmax1. Responder analysis indicated that COPM and ArmA improvements were clinically significant for up to 50% of participants. Active upper extremity use (ArmA, part B), health-related quality of life (SF-12v2), and upper extremity strength (MI) remained unchanged. **Conclusions**: Our results indicate that BoNT-A in combination with routine outpatient therapy positively influenced the individually valued daily activities of stroke survivors. COPM, GAS, and ArmA are suitable for facilitating a patient-centred and daily life-oriented spasticity management post-stroke.

## 1. Introduction

During daily life, stroke survivors with upper extremity spasticity and their carers are confronted with stiff muscles, resisted or even restricted passive range of motion, and joint deformities, which in turn may, for example, cause pain and difficulties during dressing and personal hygiene, skin maceration, and hamper reaching for objects as well as grasping and releasing them actively [1]. As a consequence, daily activities can become demanding, time-consuming, and frustrating [2].

A recommended therapeutic option for treating spasticity is the injection of botulinum toxin type A (BoNT-A) [3,4]. Robust evidence demonstrates its efficacy in reducing resistance to passive movement, especially for wrist and finger flexors, and in improving the ability to care for the affected upper extremity [5]. Significant positive effects are also reported for spasticity-related upper extremity pain, passive range of shoulder motion, associated reactions, and caregiver burden [5].

However, despite these benefits, a specific gap remains: the extent to which routine BoNT-A treatment influences meaningful, patient-/carer-identified daily activities within real-world environments is less well understood. Prior research has predominantly emphasized clinical impairments, such as muscle tone or joint mobility, while giving limited consideration to meaningful activities, patient/carer experiences, and individual needs defined by the patient/carers themselves [5,6]. Evidence from controlled studies often fails to capture outcomes that occur within the contextual realities of home and community life. Although goal-oriented assessment has been addressed in some previous studies, emphasis has often been placed on impairment-level outcomes or standardized functional measures, with comparatively limited focus on activities.

Consequently, evidence remains limited regarding the influence of routine BoNT-A treatment on the lived experience of daily activities. Likewise, the understanding of how goal-oriented and patient-centred approaches are translated into everyday clinical contexts remains limited. Thus, there is a need to examine BoNT-A therapy beyond standardized research environments, addressing patient perspectives as determinants of satisfaction and real-world functional impact and integrating practical aspects of treatment delivery.

To address this gap, we adopted an explicitly patient-centred and occupation-focused approach. Occupations can be defined as activities that provide meaning, structure, and purpose in daily life. They include the range of tasks an individual needs, wishes, or is expected to perform, such as work, leisure, domestic responsibilities, and self-care [7]. Given their inherently individualized nature, occupations offer a sensitive framework for understanding personal priorities, perceived difficulties, goal setting, and contextual influences. We therefore prioritized assessments at the ‘activities and participation’ levels of the International Classification of Functioning, Disability and Health (ICF) [8]. However, we also incorporated objective as well as quality of life outcome measures and an assessment performed at the ICF level of ‘body functions’ to ensure a holistic view [5,9,10], without shifting the emphasis away from activity-oriented, patient-rated outcomes. We paid particular attention to practical considerations regarding the application of the assessments, as these aspects are crucial for integrating patient-centred assessment effectively into routine clinical practice.

Therefore, the objective of this study was to evaluate the individualized, real-world benefits of routine outpatient BoNT-A management for stroke survivors with upper extremity spasticity through the implementation of a patient-centred, daily life-oriented, and holistic approach. The study was intended to facilitate a BoNT-A treatment strategy focused on individual patient/carer needs for the performance of concrete meaningful daily activities and to evaluate the suitability of different outcome measures for their use in routine clinical practice.

## 2. Materials and Methods

### 2.1. Eligibility Criteria and Study Procedures

This observational study was performed in a spasticity outpatient clinic. Ethical approval was obtained from the local ethics committee (Universitätsmedizin Greifswald).

All adult patients (≥18 years) attending the outpatient clinic to receive BoNT-A injections for upper extremity spasticity following stroke (ischemic stroke, intracerebral or subarachnoidal haemorrhage) were eligible to participate in the study. There were no exclusion criteria relating to chronicity of stroke, number of BoNT-A injections in the past, or additional antispastic medication.

Written informed consent was obtained from patients or their legal representatives, with written assent from the patient where possible.

In line with standard clinic care, participants received BoNT-A from one of two experienced neurologists. Muscle selection, BoNT-A brand, and dosage were individualized based on clinical presentation and treatment goals. Injections were guided by electromyography and electrical stimulation.

Participants also completed a structured interview and assessment with an occupational therapist experienced in stroke rehabilitation. Data were collected over one injection cycle (12–14 weeks) on three study visits. The first (T0) and third (T2) visits coincided with routine BoNT-A treatment appointments, with study procedures scheduled beforehand. The second visit (Tmax1), at peak drug efficacy [11], occurred 4–6 weeks post-injection. If participants could not attend the clinic (for Tmax1) or had limited time during T0 or T2, some procedures were offered at home, by phone, or in the hospital.

### 2.2. Baseline Characteristics and Treatment Information

Participants’ age, sex, time since stroke, degree of disability (Oxford Handicap Scale, OHS [12]), BoNT-A injection details, and type and frequency of physio-and/or occupational therapy during the study were documented.

### 2.3. Outcome Measures

#### 2.3.1. Primary Outcome Measures

We used three primary outcome measures, all reflecting the ICF level activities and participation.

The Canadian Occupational Performance Measure (COPM) [13] was chosen to identify the most important (up to five) individual everyday occupational performance issues for BoNT-A treatment by means of a semi-structured interview at T0. The current baseline performance of the selected daily activities and the level of satisfaction with their performance were also evaluated from the participants’ and/or carers’ perspective and re-assessed 4 to 6 and 12 to 14 weeks later (Tmax1 and T2, respectively). A scale ranging from 1 to 10 (performance: 1 = ‘extremely poor/cannot do’, 10 = ‘can do extremely well’; satisfaction: 1 = ‘not satisfied at all’, 10 = ‘extremely satisfied’) was employed.

Goal Attainment Scaling (GAS) [14] was used to select one individual meaningful daily activity at T0 as a primary goal for treatment. For these activities/goals, six graded observable and SMART (specific, measurable, achievable, realistic/relevant, timed) [15] behaviour levels were developed. Goal attainment was rated at Tmax1 and T2 using the previously defined individual 6-point GAS scale, where a score of 0 implies ‘the goal has been attained as expected’, +1 = ‘somewhat more than expected’, +2 = ‘much more than expected/best possible outcome for the selected goal’, −1 = ‘less than expected/the participant has improved but did not fully attain the goal’, −2 = ‘no change from baseline’, and −3 = ‘worsening compared to baseline’.

The Arm Activity Measure (ArmA) [16,17] formed the basis for a structured interview to evaluate the (passive) care activities of the hemiparetic arm (part A) and active use of the affected upper extremity (part B) within participants’ usual settings and situations over the preceding seven days. This measure was developed specifically for patients receiving focal spasticity treatments. Accordingly, the amount of difficulty for the eight passive and thirteen active function items (activities) was rated by participants and/or carers at T0, Tmax1, and T2 using a 5-point Likert scale, where 0 points refers to ‘no difficulty’ and 4 points to ‘unable to do activity’.

We also calculated responder rates (i.e., the proportion of participants demonstrating a clinically significant response) for the three primary outcome measures. Participants were categorized as responders if they obtained a difference of ≥2 points for the COPM [18], a GAS score of ≥0, or an improvement of 3.0 points for part A of the ArmA or 2.5 points for part B [17].

#### 2.3.2. Secondary Outcome Measures

The Resistance to Passive Movement Scale (REPAS) [19] is a summary rating scale based on the Ashworth Scale [20], a measure at the ICF level of body functions. It consists of a set of standardized arm (part A) and leg (part B) assessments, each using a 5-point Likert scale, where 0 points represents ‘no increase in muscle tone’ and 4 points ‘limb rigid in flexion or extension’. We used the REPAS to evaluate resistance to eight full-range passive upper extremity joint movements.

The Motricity Index (MI) [21,22] was deployed at T0, Tmax1, and T2 to document the degree of paresis/strength at the ICF level of body functions. Two 6-point Likert scales, ranging from 0 = ‘no movement’ to 33 = ‘normal pinch grip’/‘normal power’, were applied for three upper and lower extremity movements.

The SF-12v2 Health Survey (SF-12v2) [23] is a questionnaire comprising 12 items to address health-related quality of life, a measure at the ICF level of activities and participation. Items are assessed with 5- or 3-point Likert scales. A physical component summary (PCS) and a mental component summary (MCS) are calculated, each ranging between 0 (indicating the lowest self-reported health-related quality of life) and 100 points. The acute version (1-week recall) was used during all three study visits.

The treatment benefit of the BoNT-A injection, i.e., the patient’s condition in comparison to the condition at admission to the project, was assessed by participants/carers and investigators separately using Global Clinical Impression (GCI) at Tmax1 and T2. GCI was also collected from treating outpatient therapists for Tmax1. We applied a 4-point Likert scale, ranging from 1 = ‘very good’ to 4 = ‘bad’ [24]. When more than one rating was available, for example, from both the participant and their carer, the average was used for analysis.

Visual Analogue Scales (VAS) were employed to document the importance of and satisfaction with the BoNT-A treatment from the participants‘/carers’ perspective at T2. We used a horizontal line without markings, 100 mm in length, with the boundaries ‘not at all important’/‘not at all satisfied’ and ‘most important’/‘most satisfied’.

### 2.4. Data Analyses

Data were transferred to MS Excel and analysed using IBM SPSS Statistics (versions 23 and 24). The significance level was set at 0.05. Nonparametric statistics were applied to account for the ordinal nature of the outcome measures and the relatively small sample size. Here, nonparametric tests are considered robust without requiring that normal distribution or variance homogeneity assumptions are met. The Friedman test was used for those measures with three measurement time-points (COPM, ArmA, MI, REPAS, and SF12v2). Wilcoxon signed-rank tests were used for pairwise comparisons (with Bonferroni correction) in cases of statistical significance and for GAS and GCI. Statistical analyses excluded those participants with incomplete data for the respective outcome measure. T-scores were calculated for SF12v2 using a suggested validated algorithm [23].

## 3. Results

### 3.1. Participants

**Figure 1:**
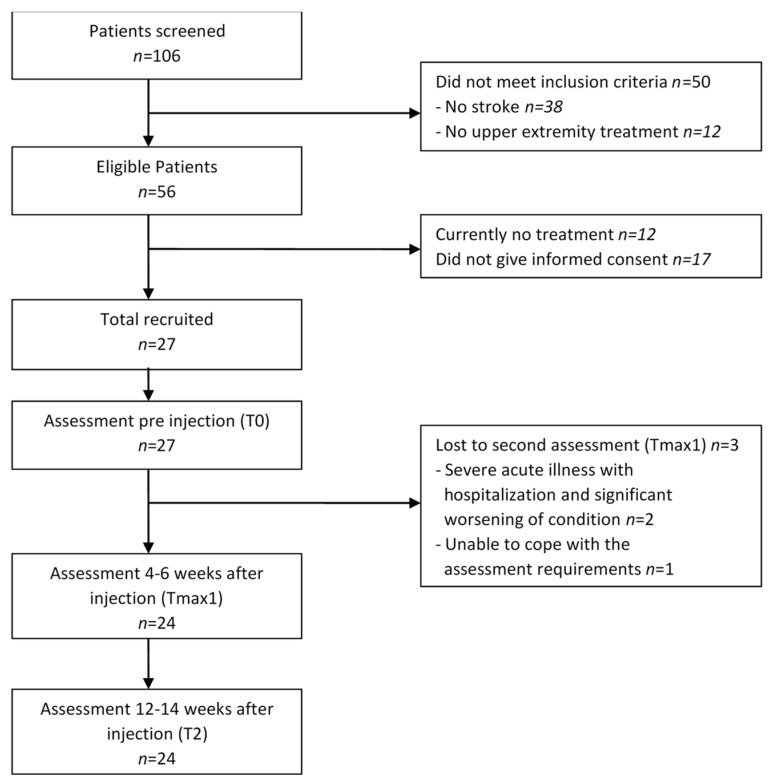
Patient flow over the study period.

All patient records within our outpatient spasticity clinic were screened. Twenty-seven eligible patients (or their legal representatives) consented to participate (Figure 1).

The mean age of participants was 58.2 years (SD 12.0; range 34–82 years); 59.3% were male. The mean period since (first) stroke was 7.1 years (SD 5.3); 59.3% participants had left-side and 40.7% right-side paresis. According to the OHS, all except one of the participants were moderately to severely disabled and required assistance; three of these were totally dependent on others.

### 3.2. Treatment (Botulinum Toxin A Injections, Physiotherapy, and Occupational Therapy)

Participants were injected with BoNT-A consistent with usual practice. Between two and eight upper extremity muscles were chosen for injection per individual (mean 4.9 muscles, SD 1.6). Details for the BoNT-A treatment at baseline (T0) are shown in Table 1.

All participants with data over one injection cycle (*n* = 24) received routine outpatient physiotherapy (PT) and/or occupational therapy (OT): PT, 95.8% (*n* = 23); OT, 70.8% (*n* = 17); both, 66.7% (*n* = 16). Each session typically lasted between 30 and 45 min. One individual participated in inpatient PT and OT during a period of hospitalization between Tmax1 and T2. The remaining participants were engaged between T0 and T2 in a mean number of 34.9 (SD 11.5, range 15–59) outpatient therapy sessions (PT: mean 22.8, SD 6.4, range 9–35; OT: mean 17.7, SD 7.0, range 7–32 sessions).

### 3.3. Goal Attainment Scaling

**Figure 2:**
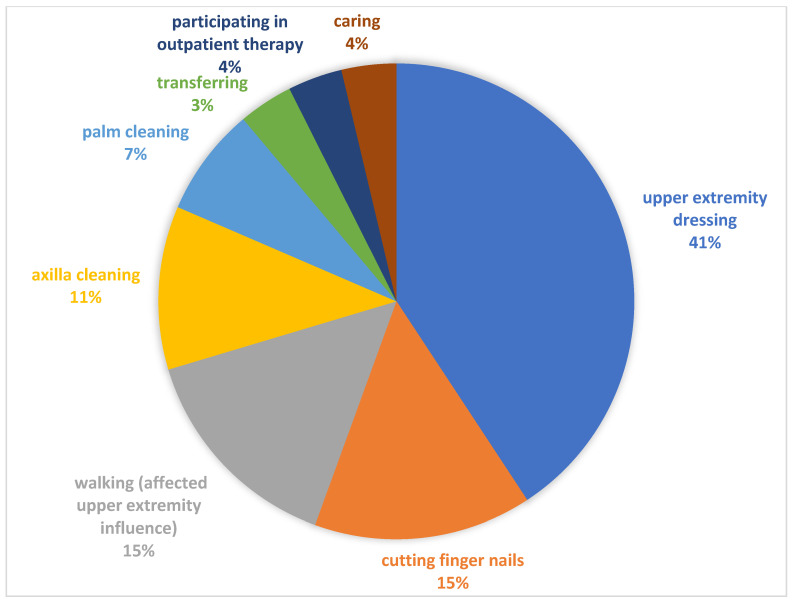
Categories of identified goals using GAS.

Each participant/carer set one specific activity-based goal for BoNT-A treatment during T0 using GAS. The set goals were categorized into eight activity areas (see Figure 2).

GAS scores four to six weeks after injection (Tmax1) improved in 18 participants (75.0%), with goals fully achieved by 8 participants (33.3%), while 6 participants (25.0%) experienced no change. Twelve to fourteen weeks after injection (T2), 16 participants (66.6%) still showed an improvement, of whom 3 (12.5%) achieved or slightly over-achieved (*n* = 1) their set goal. Seven participants (29.2%) showed no change, while worsening was experienced by one (4.2%) participant. There was no statistically significant difference between goal attainment at Tmax1 and T2 (*p* = 0.124).

### 3.4. Canadian Occupational Performance Measure

Problems relating to occupational performance in the three areas of self-care, productivity, and leisure were identified by all participants/carers. They then rated their importance to them and selected up to four (mean 2.2, SD 0.9) of the most important problems relevant to BoNT-A treatment. Performance of these occupations, as well as satisfaction with performance, improved significantly from T0 to Tmax1 and from T0 to T2, while no significant difference between Tmax1 and T2 was observed (Table 2).

### 3.5. Arm Activity Measure

After BoNT-A treatment, participants/carers reported fewer difficulties during care of the affected upper extremity (passive function subscale). This effect was significant in the T0 to Tmax1 and the T0 to T2 phases, with no effect between Tmax1 and T2 (Table 2).

The active function subscale indicated that almost all participants had remarkable difficulties or were not able to use and manipulate objects with their affected upper extremity. Over time, the median sum scores and IQR for active function were relatively stable. While the Friedman test revealed a significant effect, this was not apparent following post hoc analyses (Table 2).

### 3.6. Secondary Outcomes

Three participants (11.1%) used the telephone option across the whole study, and one did so for T2; therefore, assessment data requiring physical contact (MI and REPAS) could not be collected for these instances.

Details for the results of the secondary outcome measures are depicted in Table 2.

Resistance to selected upper extremity movements (REPAS) showed a similar pattern to that already observed for COPM and ArmA, with significant improvements from T0 to Tmax1 and T0 to T2, but no significant change between Tmax1 and T2.

Upper extremity strength, as measured by MI, was not influenced by the BoNT-A treatment.

Similarly, quality of life (SF-12v2) did not change over the injection cycle, either for physical or mental health. When the participants’ scores were compared to the U.S. norm with a mean score of 50.0 and a SD of 10, it showed that mental health scores were broadly comparable (mean (SD): T0, 47.6 (8.6); Tmax1, 49.7 (10.3); T2, 49.3 (12.2)), while physical health was markedly reduced (mean (SD): T0: 33.4 (7.9); Tmax1, 36.0 (7.7); T2, 34.7 (7.1)).

BoNT-A treatment benefit (GCI) ratings were obtained for all 24 participants completing the study with full data from participants/carers, study personnel, and outpatient therapists. Ratings ranged from “very good” to “bad”. The median scores for participant/carer as well as physician/therapist estimated both at Tmax1 and T2 correspond to a “good” treatment benefit (median 2.0), while outpatient therapists showed a somewhat more critical but still positive view (median 2.5). A “bad” treatment benefit was observed eight times (Tmax1: *n* = 5, T2: *n* = 3) and pertained to six participants. Ratings did not differ significantly between Tmax1 and T2 (*p* = 0.248).

For most participants/carers, BoNT-A treatment was shown to be of importance, and most ratings for satisfaction with treatment were also at the higher end of the VAS (importance—median 74.0 mm, IQR 55.5–89.5; satisfaction—median 75.0 mm, IQR 60.3–91.5).

### 3.7. Responder Analyses (i.e., Participants Experiencing a Clinically Significant Response)

**Figure 3:**
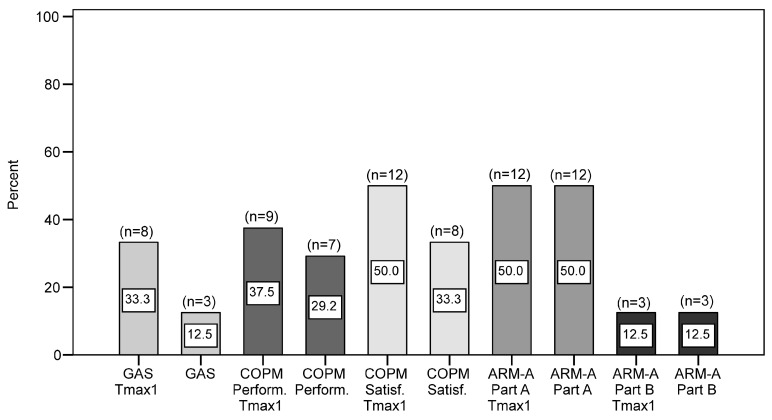
Clinically significant response rates at Tmax1 and T2 for GAS, COPM, and ARM-A (*n* = 24). GAS: Goal Attainment Scaling; COPM: Canadian Occupational Performance Measure; Perform.: Performance subscale; Satisf.: Satisfaction subscale; ARM-A: Arm Activity Measure; part A—passive function subscale; part B—active function subscale.

Responder rates for GAS, COPM (performance and satisfaction, respectively), and ArmA (active and passive function) illustrate the proportion of participants with a clinically significant treatment result (Figure 3). Such improvement was obtained by 12.5% to 50.0% of participants, at both Tmax1 and T2, with the highest responder rates for COPM satisfaction and ArmA passive care.

## 4. Discussion

This observational study provides insights into the process and outcomes in relation to the management of stroke survivors with upper extremity spasticity. It shows how a routine BoNT-A injection in combination with outpatient therapy improves the performance of daily activities and satisfaction with the performance of these activities, in addition to any reduction in the resistance to passive movement. However, effects for active arm/hand use and strength, as well as health-related quality of life, were limited. Despite these limitations, BoNT-A treatment was considered important by most participants/carers. The explicit identification of patient-centred and occupation-focused goals for the BoNT-A treatment, the assessment of goal achievement with outcome measures that specifically capture such benefits, and a responder analysis all indicate that not only statistical, but clinically meaningful changes were induced.

In addition, the results support the notions that the patient-centred and occupation-focused approach can be implemented in routine healthcare, is valued by stroke survivors, and promotes an individually meaningful integration of BoNT-A treatment in spasticity management.

With regard to the assessment tools selected for this research, the main outcome measures (i.e., COPM, GAS, and ArmA) were shown to be valuable for a BoNT-A assessment and treatment focusing on the meaningful everyday needs of stroke survivors with upper extremity spasticity.

This study followed a cohort of patients receiving BoNT-A treatment as part of routine management and applied an individual, holistic, and person-centred assessment approach as recommended by Turner-Stokes et al. [25]. The aim was to optimize patient management by focusing on the individual daily life challenges experienced by participants/carers and on related outcome measures. The latter were selected to capture the ICF components ‘activities and participation’ and ‘body functions’ and were applied within individual ‘environments’ (usual homes and ordinary life situations). Objective assessment was enhanced by including patient-reported information and complemented by a quality-of-life assessment to maintain a holistic view of participants’ lives, as recommended for evaluation [9,10]. Such a holistic approach is rather unusual within spasticity management and research, where the focus on clinical signs and symptoms is typical, though some have been critical of this approach [6,25]. Another strength of our study was the timeframe used for evaluation, which included the observation of the full treatment effect (i.e., four to six weeks after injection; Tmax1). While patients usually return to treatment and assessment when the effect of the drug is waning (often at least three months after injection), this adds to a more complete picture. And lastly, to our knowledge, this is the only study in the field to date that includes views from treating therapists from the community, an important group of healthcare professionals involved in spasticity management.

We used three main outcome measures in our study: GAS, COPM, and ArmA. The GAS is specifically suggested as an outcome measure for spasticity management [25] and is widely applied within studies evaluating the effect of BoNT-A for patients with upper extremity spasticity [26,27,28,29,30,31,32,33,34,35]; patients value being actively involved in the goal-setting process [36,37]. While setting one’s own goals may lead to greater autonomy and satisfaction [36], increased motivation, and reduced anxiety of lay carers [37], the use of GAS may be time-consuming [38,39,40], and defining distinct and SMART goal levels can be difficult [41]. We had similar experiences, but the predefined SMART and distinct goals made subsequent goal evaluation fast and simple. For routine spasticity assessment, the ‘GAS-light’ model [42] may provide an effective alternative, since it was specifically developed to overcome some of the above-mentioned limitations.

The use of the COPM before goal setting with GAS in our study helped us to shorten the goal identification process with GAS considerably. When asked, about half of our participants/carers could not spontaneously identify any spasticity-related challenges during daily life. Similar difficulties were also observed by Law et al. [43]. Using the COPM allowed us to collect individual difficulties in the three main life areas of self-care, productivity, and leisure in a client-centred and structured manner. While problem identification required considerable time, a point also noted by Toomey, Nicholson, and Carswell [44], subsequent re-evaluation was relatively efficient.

While COPM has been shown to be a valuable assessment tool within routine spasticity management, administration requires a fair amount of time and specialist knowledge of the Canadian Model of Occupational Performance and Engagement [45]. Optimal practice typically requires a regular exchange of information between the injecting staff and therapists to assess, coordinate, adapt, and optimise both BoNT-A and therapeutic treatment.

Finally, our third main outcome measure, ArmA, comprises a range of relevant pre-selected activities. It could be filled in by the patients/carers themselves, and it was relatively easy and quick to use in our study. Most of our participants preferred this outcome measure, although part B, which relates to active function, was partly regarded as being too difficult; this was also reported by ~1/4 of the sample in Ashford et al. [46]. However, from our experience, the ArmA constitutes a suitable outcome measure for use within outpatient BoNT-A management.

When focusing on the results obtained by the implemented assessments in our study, they were largely in accordance with those of previous systematic reviews/randomized controlled trials (RCTs). This applies to both functional disability (in chronic stroke survivors) and active upper extremity function [5,46,47], resistance to passive movement [5,47,48,49], and health-related quality of life (QOL) over one injection cycle [47,50,51,52]. The results are also supportive of previously reported disability responder rates [53]. Observational studies have shown similar results for ease of care/functional disability [27,28] and resistance to passive upper extremity movement [27,28,33,54].

According to our results and those found in the literature, it seems that improved passive joint movement may directly facilitate care tasks, such as putting the affected arm through a sleeve during dressing or washing one’s hands, where the upper extremity is passively involved. However, a reduction in muscle tone and related stiffness seems less likely to improve the performance of those tasks where the affected upper extremity is actively involved and to affect QOL results. There are several factors that may account for this. Firstly, it may be necessary to follow up with patients over extended periods with repeated BoNT-A administrations (at least four injections) to be able to observe an effect [33,55]. Secondly, the subgroup of patients with a degree of preserved motor control, receiving both repeated BoNT-A injections and rehabilitative therapy, may have a greater potential to improve the execution of active tasks, as Levy et al. [56] hypothesize. Thirdly, the lack of a QOL effect after just one injection may be associated with the generic nature of QOL scales and the subsequent diminished sensitivity for detecting change [51]. Such changes would be more likely with more BoNT-A intervention cycles and/or time for re-evaluation. Accordingly, improvement in QOL has been observed in studies that repeatedly administered injections [28,50,54,57]. Fourthly, improvements in active arm and hand use as well as QOL may be more complexly inter-related, with contributing factors including exercise and movement therapy, coping strategies, environmental factors, and compensation, as well as cumulative BoNT-A effects.

In contrast to the largely homogeneous findings in the literature regarding resistance to passive movement, functional disability, active upper extremity function, and QOL over one injection cycle, goal attainment rates differ considerably in the literature (e.g., 23% in Turner-Stokes et al. [58] vs. 94% in Ashford and Turner-Stokes [27]). Study aspects such as the goal-setting process, the BoNT-A treatment used, and the nature of the sample involved vary and may be factors that contribute to the different rates reported. It should be noted that those studies achieving high responder rates often used impairment-based goals such as pain reduction or improvement in range of motion [27,28,35]. In contrast, participant goals in this study focused on the ICF component ‘activities and participation’. (Full) goal attainment in our study was relatively limited at 33%. Turner-Stokes et al. [58] observed even smaller attainment rates between 3% and 12% for those goals set at the ICF ‘activities and participation’ levels. Adding to these findings, a large-scale RCT, one of the few studies in the field that included the COPM, did not reveal an effect from BoNT-A for the performance of individual occupations, which can be assigned to the ICF component ‘activities and participation’, or for satisfaction with their performance [52]. The limited effect at the ‘activities and participation’ level may be explained by the mode of action of the drug, which can be considered to occur at the impairment level. Surprisingly, Nott et al. [32] found no differences in goal achievement as a function of ICF level. In this study, set goals were linked to the ICF levels and examined for potentially influencing factors for goal attainment. It should be noted that their sample was small, and their participants’ upper extremity function was less impaired. Further factors may potentially influence goal achievement, such as the number of BoNT-A injections [59], initiation of treatment [60], and the proportion of goals requiring active arm and hand movements [34].

### Study Limitations

There are a number of limitations to this study. Firstly, it was conducted in a single centre, and the number of participants was relatively small. These features limit generalizability, as the patient population and care protocols in this setting may differ from those in other clinics or regions. Secondly, compared to standard care elsewhere, our participants had received a relatively high amount of physio- and occupational therapy. It is plausible that the combined effect of BoNT-A treatment and regular physiotherapy, and frequently also occupational therapies, resulted in greater improvements than would have been achieved by BoNT-A alone. This multimodal therapy, while reflective of routine care in this setting, may limit the applicability of the results to other contexts. Thirdly, an inherent disadvantage of observational studies is the lack of a control group. Fourthly, only one BoNT-A injection cycle was observed, while it is possible that some changes may only be observed after multiple injections [33,55]. Fifth, participants had already received BoNT-A in the past, which may have led to an underestimation of the effect, if a certain reduction in spasticity from the previous administration was still present at injection (T0). Lastly, while patient-reported outcome measures are an integral part of a patient-centred approach, difficulties in differentiating between spasticity and paresis and in remembering accurately may limit their validity.

Variations in outpatient physiotherapy and occupational therapy, BoNT-A treatment parameters, and patient characteristics represent potential confounding (or at least modifying) factors noted in the limitations and underscore the complexity of the clinical context (i.e., routine healthcare). The fact that despite such contextual variability, the patient-centred and occupation-focused integration of BoNT-A treatment in spasticity management induced personally valued and clinically meaningful improvements in a considerable proportion of participants nevertheless supports its usefulness.

We recommend focusing future studies on the collaboration of healthcare providers to improve assessment and treatment to support patients and caregivers in managing daily life issues. Combining a daily living- and patient-centred approach with an earlier treatment initiation [60,61,62] and the use of higher doses of BoNT-A [63,64,65,66,67,68] over a study period of at least 12 months with multiple injection cycles may warrant further investigation. This would allow the analysis of cumulative and sustained effects of treatment, providing deeper insights into optimizing patient outcomes in everyday life. Other potentially influential factors, such as the subjective importance patients attribute to BoNT-A treatment and the role of hopes and beliefs, could merit further investigation to better understand their impact on treatment outcomes.

## 5. Conclusions

In conclusion, this observational study has shown that patient-centred, holistic, and everyday life-oriented assessment following a process structure that allows stroke survivors (and their caregivers) to identify and assess meaningful occupational performance problems and goals for BoNT-A treatment is possible, but this approach may be challenging and time-consuming. BoNT-A in combination with outpatient occupational and/or physiotherapy clearly has the potential to positively impact daily living, measurable in terms of reducing activity limitations and participation restrictions, and being clinically relevant for at least a proportion of treated patients.

## Figures and Tables

**Table 1:** Doses used per respective agent and muscle, with number of participants injected (*n* = 27).

Muscles	Number of Participants (*n*)	Type of BoNT-A	Units	
			Mean (SD)	Range
Biceps brachii	10 (37.0%)	Incobotulinum toxin A (*n* = 7)	32.9 (9.5)	(20–50)
		Onabotulinum toxin A (*n* = 3)	36.7 (15.3)	(20–50)
Brachialis	14 (51.9%)	Incobotulinum toxin A (*n* = 12)	29.2 (5.1)	(20–40)
		Onabotulinum toxin A (*n* = 2)	40.0 (0.0)	(40–40)
Brachioradialis	1 (3.7%)	Onabotulinum toxin A (*n* = 1)	20	
Deltoideus	2 (7.4%)	Incobotulinum toxin A (*n* = 2)	30.0 (0.0)	(30–30)
Extensor digitorum communis	1 (3.7%)	Incobotulinum toxin A (*n* = 1)	30.0	
Flexor carpi radialis	14 (51.9%)	Incobotulinum toxin A (*n* = 13)	28.9 (8.7)	(20–50)
		Onabotulinum toxin A (*n* = 1)	20	
Flexor carpi ulnaris	6 (22.2%)	Incobotulinum toxin A (*n* = 6)	25.0 (8.4)	(20–40)
Flexor digitorum profundus	20 (74.1%)	Incobotulinum toxin A (*n* = 15)	25.0 (6.8)	(15–40)
		Onabotulinum toxin A (*n* = 4)	22.5 (5.0)	(20–30)
		Abotulinum toxin A (*n* = 1)	200	
Flexor digitorum superficialis	18 (66.7%)	Incobotulinum toxin A (*n* = 15)	47.3 (12.2)	(30–70)
		Onabotulinum toxin A (*n* = 2)	40.0 (0.0)	(40–40)
		Abotulinum toxin A (*n* = 1)	300	
Flexor pollicis brevis	2 (7.4%)	Incobotulinum toxin A (*n* = 2)	5.0 (0.0)	(5–5)
Flexor pollicis longus	7 (25.9%)	Incobotulinum toxin A (*n* = 4)	12.5 (5.0)	(10–20)
		Onabotulinum toxin A (*n* = 2)	10.0 (0.0)	(10–10)
		Abotulinum toxin A (*n* = 1)	100	
Lumbricales	1 (3.7%)	Incobotulinum toxin A (*n* = 1)	15.0	
Opponens pollicis	3 (11.1%)	Incobotulinum toxin A (*n* = 3)	8.3 (2.9)	(5–10)
Pectorales	15 (55.6%)	Incobotulinum toxin A (*n* = 14)	31.1 (7.9)	(20–40)
		Onabotulinum toxin A (*n* = 1)	40	
Pronator teres	10 (37.0%)	Incobotulinum toxin A (*n* = 10)	28.0 (6.3)	(20–40)
Triceps brachii	7 (25.9%)	Incobotulinum toxin A (*n* = 7)	37.1 (19.8)	(20–70)

**Table 2:** Summary of outcome measure scores and related statistical findings across the study time-points.

Outcome Measure	T0 (Baseline)	Tmax1 (4–6 Weeks)	T2 (12–14 Weeks)	*p*-Value *	*p*-Value †		
					T0–Tmax1	T0–T2	Tmax1–T2
COPM (*n* = 24)							
Performance	4.3 (3.0–6.0)	6.0 (4.5–7.7)	5.3 (4.6–7.0)	<0.0001	<0.001	<0.004	0.052
Satisfaction	3.8 (2.6–5.9)	6.0 (4.4–7.5)	5.7 (4.0–7.3)	<0.0001	<0.001	<0.001	0.129
ArmA (*n* = 24)							
Passive function	12.5 (6.0–16.0)	7.0 (4.3–10.8)	9.5 (5.0–12.0)	<0.0001	<0.0001	0.001	0.196
Active function	37.0 (30.3–47.3)	36.5 (30.0–47.0)	36.0 (30.3–46.5)	0.011	0.020	0.586	0.197
REPAS (*n* = 21)	9.0 (7.0–12.0)	7.0 (4.5–9.5)	8.0 (4.5–10.0)	<0.001	0.002	0.001	0.054
MI (*n* = 21)	40 (26.5–56.0)	40 (34.5–61.0)	40 (29.0–59.0)	0.157	-	-	-
SF-12v2 (*n* = 24)							
PCS	35.5 (28.0–38.8)	37.2 (31.3–41.0)	33.3 (31.8–39.1)	0.180	-	-	-
MCS	47.8 (43.6–53.4)	51.0 (44.6–57.2)	50.7 (41.8–57.4)	0.819	-	-	-
GCI (*n* = 24)							
Participant/carer	-	2.0 (2.0–2.4)	2.0 (2.0–2.5)	-	-	-	0.286
Physician/therapist	-	2.0 (2.0–3.0)	2.0 (2.0–3.0)	-	-	-	0.248
Outpatient therapist	-	2.5 (2.0–3.0)	-	-	-	-	-
VAS (mean (SD)) (*n* = 24)							
Importance	-	-	70.2 (22.6)	-	-	-	-
Satisfaction	-	-	73.6 (18.2)	-	-	-	-

Data are represented as median (IQR), unless otherwise stated. IQR: interquartile range; * Friedman test; † (Post-hoc) Wilcoxon signed-rank tests (Bonferroni corrected with new significance level set at *p* < 0.017). COPM: Canadian Occupational Performance Measure (scale ranges 1–10); ArmA: Arm Activity Measure, passive function subscale (sum score, range 0-28), active function subscale (sum score, range 0–52); REPAS: Resistance to Passive Movement Scale, upper extremity part (sum score, range 0–32); MI: Motricity Index, upper extremity part (sum score, range 1–100); SF12v2: SF-12v2 Health Survey, PCS: physical component summary, MCS: mental component summary. GCI: Global Clinical Impression (score, range from 1 = very good to 4 = bad). VAS: Visual Analogue Scale (scale, range 0–100 mm) for importance of the BoNT-A treatment and satisfaction with treatment rated by participants/carers.

## Data Availability

The data that support the findings of this study are available by contacting the corresponding author via e-mail.

## Abbreviations

The following abbreviations are used in this manuscript:

ArmA	Arm Activity Measure
BoNT-A	Botulinum toxin type A
COPM	Canadian Occupational Performance Measure
GAS	Goal Attainment Scaling
GCI	Global Clinical Impression
ICF	International Classification of Functioning, Disability and Health
MI	Motricity Index
OHS	Oxford Handicap Scale
REPAS	Resistance to Passive Movement Scale
SF-12v2	SF-12 Health Survey, version 2
VAS	Visual Analogue Scale
